# Editorial: Microbial mitigation of hazardous compounds in agro-ecosystems

**DOI:** 10.3389/fmicb.2022.1015111

**Published:** 2022-10-13

**Authors:** Devanita Ghosh, David E. Whitworth, Hendrik Schäfer, Srinivasan Krishnamurthi, Pradipta Saha

**Affiliations:** ^1^Laboratory of Biogeochem-Mystery, Centre for Earth Sciences, Indian Institute of Science (IISc), Bangalore, India; ^2^Sanitary Engineering Section, Water Management Department, Technische Universiteit (TU) Delft, Delft, Netherlands; ^3^Institute of Biological, Environmental and Rural Sciences, Aberystwyth University, Ceredigion, United Kingdom; ^4^School of Life Sciences, University of Warwick, Coventry, United Kingdom; ^5^Microbial Type Culture Collection & Gene Bank (MTCC), CSIR-Institute of Microbial Technology (IMTECH), Chandigarh, India; ^6^Department of Microbiology, The University of Burdwan, Burdwan, India

**Keywords:** biodegradation, bioremediation, agro-ecosystems, heavy metals, xenobiotic

Currently, many agro-ecosystems are contaminated with toxic, hazardous compounds which can be broadly categorized as (a) anthropocentrically introduced xenobiotic agrochemicals (e.g., pesticides, insecticides, and fungicides), (b) secondary metabolites produced as a result of plant-microbe interactions, and (c) heavy metals which are mainly introduced through natural and anthropogenic processes. Organophosphates (OP), carbamates, pyrethroids, and neonicotinoids are among the most dominant groups of xenobiotic agrochemicals reported globally. These are highly toxic to non-target organisms including humans and many have been banned by the US Environmental Protection Agency or the European Union. Among these, neonicotinoids were introduced recently (in the 1990s), being comparatively less toxic to non-target higher vertebrates and most extensively used (149 crops in 120 countries). An example of a naturally produced hazardous compound is Deoxynivalenol (vomitoxin, DON), which is a highly toxic secondary metabolite (mycotoxin) produced by the mold *Fusarium* while infecting staple crops. Finally, Pb, Cr, Cd, As, Zn, Cu, and Fe are commonly encountered heavy metals in agroecosystems globally.

Although toxic compounds are applied to protect against pests and diseases and thereby increase crop yields, ironically, their extensive application has resulted in environmental pollution. Furthermore, these compounds are dispersed in the environment, through a range of different transport mechanisms and therefore affect a wider range of non-target organisms and ecosystems. For instance, the hydropedological characteristics of soils of agroecosystems allow their vertical movement (through leaching and percolation) into groundwater. Thus, while being apparently localized, these recalcitrant compounds are distributed globally and widely reported to have entered the global food web, posing a worldwide threat to human health and demanding urgent mitigation approaches to be taken. Currently, microbial mitigation strategies for hazardous compounds have the promise to be effective, highly-efficient, pollution free, reliable, and eco-friendly for agroecosystems.

Ahmed et al. provide an exhaustive review highlighting microbial groups biodegrading neonicotinoid insecticides. These groups include microalgal spp. Like *Ulothrix* sp., *Oocystis* sp., *Synechocystis* sp., *Nannochloropsis* sp.; microbe-duckweed (*Lemna turionifer* & *Ceratophyllum demersum*) combination; monoculture & mixed microalgal-bacteria culture; bacteria (*Ochrobactrum anthropi, Stenotrophomonas maltophila, Hymenobacter latericoloratus* CGMCC, *Bacillus alkalinitricus, Rhodococcus ruber, Enterobacter* sp. TMX13); and fungi (*Phanerochaete chrysosporium, Trichoderma atroviride* T23). They also discuss microbial neonicotinoid biodegradation pathways and the utility of immobilization techniques for the biodegradation of neonicotinoids.

Xu et al. report on the application of synthetic biology and recombinant DNA technologies to engineer *Escherichia coli* for the efficient biodegradation of methyl parathion (MP). MP is an extensively-used, hazardous, and OP-based agrochemical that is neurotoxic through the inhibition of acetyl-cholinesterase, leading to overstimulation of the cholinergic system, paralysis, and even death. In a unique approach, they select and synthesize six genes for expression in *E. coli*, viz., *opd* (encoding OP acid hydrase; catalyzing the hydrolysis of MP to PNP) from *Flavobacterium* sp. ATCC27551 and *pnpA-pnpE* (five genes for PNP to β-ketoadipate hydrolyzing enzymes) from *Pseudomonas putida*. The recombinant strain could biodegrade 96% of 1 mM MP within 20 to 24 h and 50% of 1 mM of *p*-nitrophenol (PNP) within 8 to 24 h. Although the authors suggest further studies before *in situ* bioremediation by the strain, the study is nevertheless the first of its kind.

An extensive review by Pande et al. focuses on the negative role of heavy metals (HM) in soil health, fertility, microbial viability, and agroecosystem processes, especially the mineralization of carbon and nitrogen, and soil enzyme activities (phosphatases, dehydrogenases, and ureases) due to Cd or/and Pb contamination. They also discuss the role of HM in the induction of oxidative stress, genotoxicity, deregulation of signaling pathways, and expression of antioxidative enzymes of plants, triggering microbial resistance through extracellular barriers, extracellular and intracellular sequestration, active transport of metal ions, and enzyme detoxification. The authors further elaborate on current studies on microbial-bioremediation processes, including bioaccumulation, biosorption, bioleaching, and biotransformation of toxic forms to non-toxic forms. Finally, they emphasize plant-microbe-based combinatorial remediation systems, and the role of recombinant DNA technology, genetic engineering, and nanotechnology in microbe-mediated HM bioremediation in developing HM bioremediation processes and technology.

Uqab et al. report a case study from Jammu & Kashmir, in India, where cement production-related heavy metal pollution of agricultural soil affected the quality and yield of the medicinally important crop saffron (*Crocus sativus*). To carry out possible bioremediation-based clean-up of HM from these agroecosystems, they study the role of microorganisms in the sequestration of cadmium (Cd) in saffron soils. They study the seasonal load (varying between 8 × 10^3^ to 9.5 × 10^4^ CFU/g of soil) of bacteria in these soil samples and isolate 72 bacterial strains (*Bacillaceae*, 43.24%; *Enterobacteriaceae*, 21.62%; *Planococcaceae*, 8.10%; *Microbacteriaceae*, 8.10%; *Pseudomonadaceae*, 5.4%; *Staphylococcaceae*, 5.4%, and *Aeromonadaceae*, 2.7%). Seven highly active Cd-sequestration strains are identified (using molecular phylogenetics and MALTI-TOF-MS typing based on peptides derived from ribosomal proteins). Both 16S rRNA gene sequence and MALDI-TOF-MS-based identification approaches gave the same results. The strain *Klebsiella pneumonia* had the highest (900 ppm) and *Enterobacter kobei* had the lowest (600 ppm) Cd-tolerance relative to *Bacillus pumilus, Pseudomonas mandelli, P. putida, Pseudomonas avellanae* and *Staphylococcus equorum*. The authors propose the possible application of these strains for the effective detoxification and elimination of HMs from saffron fields contaminated with Cd.

Zhang et al. carry out extensive screening of soil samples collected from wheat and corn fields, and forests in China, followed by enrichment of DON-degrading bacteria, to isolate *Nocardioides* sp. ZHH-013. They find that the DON biodegradation rate was directly proportional to the amount of live cell biomass. Using HPLC and UPLC-MS/MS analyses, they identify a novel biodegradation mechanism, involving the formation of 3-*keto*-DON and 3-*epi*-DON. The strain could completely mineralize DON. They conclude by suggesting a possible application of this strain in the restoration of DON-polluted process water and the application of DON-metabolizing enzymes to contaminated food and feed materials.

Overall, the work presented in this Research Topic demonstrates how the application of a wide range of methodological approaches is improving the understanding of biodegradation of a variety of hazardous compounds with relevance to agriculture based on isolated microorganisms as well as microbial ecology and multi-omics approaches. Further advances toward functional biodegradation and bioremediation approaches are also achieved by recombinant DNA technology and synthetic biology methodologies. While applications require further development, the featured articles show how we are getting closer to future applications that will benefit human and ecosystem health.

## Author contributions

All authors listed have made a substantial, direct, and intellectual contribution to the work and approved it for publication.

## Conflict of interest

The authors declare that the research was conducted in the absence of any commercial or financial relationships that could be construed as a potential conflict of interest.

## Publisher's note

All claims expressed in this article are solely those of the authors and do not necessarily represent those of their affiliated organizations, or those of the publisher, the editors and the reviewers. Any product that may be evaluated in this article, or claim that may be made by its manufacturer, is not guaranteed or endorsed by the publisher.

